# Melatonin Pharmacokinetics Following Oral Administration in Preterm Neonates

**DOI:** 10.3390/molecules22122115

**Published:** 2017-12-01

**Authors:** Silvia Carloni, Fabrizio Proietti, Marco Rocchi, Mariangela Longini, Lucia Marseglia, Gabriella D’Angelo, Walter Balduini, Eloisa Gitto, Giuseppe Buonocore

**Affiliations:** 1 Department of Biomolecular Sciences, University of Urbino Carlo Bo, Via Saffi 2, 61029 Urbino, Italy; silvia.carloni@uniurb.it (S.C.); marco.rocchi@uniurb.it (M.R.); 2 Department of Molecular and Developmental Medicine, University of Siena, Viale Bracci, 53100 Siena, Italy; proietti3@gmail.com (F.P.); mariangela.longini@unisi.it (M.L.); giuseppe.buonocore@unisi.it (G.B.); 3 Department of Human Pathology in Adult and Developmental Age “Gaetano Barresi”—Neonatal Intensive Care Unit, University of Messina, Via Consolare Valeria 1, 98125 Gazzi Messina, Italy; lmarseglia@unime.it (L.M.); gabridangelo@alice.it (G.D.); egitto@unime.it (E.G.)

**Keywords:** melatonin, pharmacokinetics, preterm infants, immaturity, neuroprotective agents

## Abstract

Melatonin possesses potential efficacy in perinatal brain injuries, and has been proposed as adjunctive pharmacological therapy in combination with hypothermia in the clinical setting. However, the pharmacokinetics of melatonin in preterm and term newborns is still unknown. The aim of this study was to analyze the pharmacokinetics of melatonin after intragastric administration in preterm infants. Preterm newborns were enrolled 24–72 h after birth, and randomly assigned to three groups receiving a single bolus of 0.5 mg·kg^−1^ melatonin, or 3 boluses of 1 or 5 mg·kg^−1^ of melatonin at 24-h intervals. Blood samples were collected before and at selective times after melatonin administration. The half-life of melatonin in plasma ranged from 7.98 to 10.94 h, and the area under the curve (AUC) from 10.48 to 118.17 µg·mL^−1^·h^−1^. Our results indicate a different pharmacokinetic profile in premature newborns, compared to adults and experimental animals. The high peak plasma concentrations and the long half-life indicate that in the neonatal clinical setting, it is possible to obtain and maintain high serum concentrations using a single administration of melatonin repeated every 12/24 h.

## 1. Introduction

Perinatal brain damage, frequently associated with prematurity, is a major cause of acute mortality and chronic neurologic morbidity, including mental retardation, cerebral palsy, learning disability, or epilepsy, in infants and children [1]. To address this clinical emergency, there are no corresponding effective pharmacological therapies except for hypothermia, which is the only currently recognized treatment modality for neonatal hypoxic-ischemic encephalopathy [2]. Therefore, finding new pharmacological approaches to treat fetuses and newborns with brain damage is a high priority in perinatal care.

Melatonin, a naturally occurring indoleamine mainly produced by the pineal gland, is well known for regulating the circadian rhythm. Increasing evidence, however, indicates that melatonin also plays a role in the visual, reproductive, cerebrovascular, neuroendocrine and neuroimmunological systems [3].

Melatonin is emerging as an interesting drug for the treatment of several neurological disorders, including amyotrophic lateral sclerosis [4], Alzheimer’s disease [5], Parkinson’s disease [6], Huntington’s disease, multiple sclerosis and adult ischemic stroke [7]. An interesting proposed use of melatonin as drug is in the treatment of perinatal ischemic and inflammatory brain injuries. Indeed, in animal models, melatonin was particularly effective as a neuroprotective agent. Melatonin was found to reduce brain injury and its long-lasting consequences, after hypoxia–ischemia [8,9,10,11,12,13,14] and oxidative damage in immature rat brain [15]. In addition, melatonin was also found to improve the lipopolysaccharide-induced neonatal inflammation and related brain injury in rats [16], and the inflammatory reaction and cell death in the white matter of preterm and near-term fetal sheep, following umbilical cord occlusion [17,18]. The potential efficacy of melatonin as neuroprotective agent has also been reported in clinical studies. Indeed, a reduced serum concentration of oxidative stress markers was found in asphyxiated newborns receiving melatonin [19], and in neonates with respiratory distress syndrome [20] and bronchopulmonary dysplasia [21]. A recent randomized controlled pilot study showed that melatonin administration to neonates with hypoxic–ischemic encephalopathy preserved the serum concentration of superoxide dismutase, reduced the production of nitric oxide, and ameliorated brain injury [22]. These studies also showed that the use of melatonin did not result in any observable side effects, supporting the favorable pharmacological and toxicological properties observed in adult patients [23,24].

One limitation for the use of melatonin in the neonatal clinical setting is the paucity of pharmacokinetic data with different delivery methods and dosages. Currently, while melatonin pharmacokinetics has been clearly documented in adults [25], further investigation in neonates is still needed to optimize the dosage and the frequency of administration, a key step for a better assessment of the potential clinical efficacy in the perinatal setting. In adult animals and humans, melatonin half-life (*T*_1/2_) ranges from 18–35 min [26,27] to 45–90 min [25], respectively. This brief half-life highlights that melatonin should be administered at short intervals to obtain and maintain blood concentrations similar to those effective in experimental animals. Recently, Merchant et al. studied the pharmacokinetic profile after infusion of 0.1 mg·kg^−1^·h^−1^ melatonin for two hours in preterm neonates, in an attempt to obtain blood concentrations of the indoleamine comparable to those observed physiologically in the adult. They reported a higher half-life in neonates compared to adults [28]. However, since animal and human data indicate that the neuroprotective action of melatonin occurs at doses ranging from 5 to 15 mg·kg^−1^, much higher than those needed to replace the physiological values, it is essential to investigate the pharmacokinetics of melatonin in human newborns at comparable doses.

Here we report, for the first time, the pharmacokinetic profile of pharmacological doses of melatonin in preterm neonates after intragastric administration.

## 2. Results

### 2.1. Melatonin Concentration Assay Validation

Before assessing the melatonin concentration in blood samples, we verified the performance of our methodology. Due to the elevated dynamic range, we built two standard curves using the following ranges: 1–50 pg·mL^−1^ and 100–5000 pg·mL^−1^. The correlation coefficient (*r*^2^) was >0.99 for both standard curves. The lower limit of detection (LLD) was 1 pg·mL^−1^, whereas the lower limit of quantification (LLQ) was 2 pg·mL^−1^. The signal to noise ratio in our conditions was considerably greater than 10. The carryover was verified by sequential injection of double blank samples, whereas intra- and inter-assay precision was verified by analyzing six replicates of five quality control (QC) samples at three analytical runs. We found an intra- and inter-assay precision of 12.2% and 13.5%, respectively. After validation, the method was used for the determination of the concentration of melatonin in plasma samples. If a plasma concentration >5000 pg·mL^−1^ was detected, samples were diluted to ensure that we were working in the linear range. 

### 2.2. Melatonin Pharmacokinetics

The clinical data of the 15 newborns included in the study are reported in Table 1. Of the 15 studied, 13 showed basal serum melatonin concentrations in the pM range (mean 130.2 ± 36.7 pg·mL^−1^; range 4–506 pg·mL^−1^). The baseline data of patients P and Q were not available.

Melatonin plasma concentrations and pharmacokinetic parameters obtained after melatonin administration are reported in Table 2, Table 3 and Table 4.

**Figure 1:**
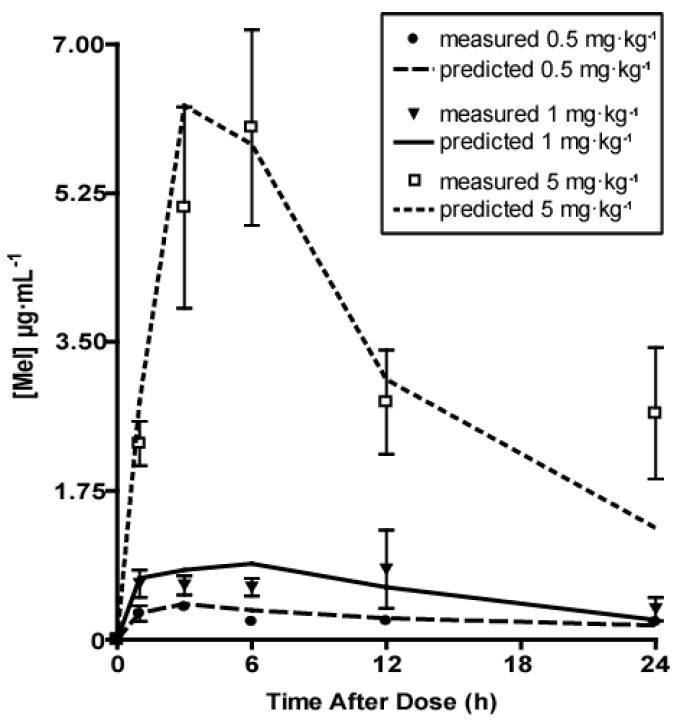
Plasma melatonin concentrations in preterm neonates after administration of an intragastric bolus. Measured melatonin (symbols) and predicted melatonin (lines) concentrations in preterm neonates receiving 0.5 (dot), 1 (triangle) or 5 (square) mg·kg^−1^ melatonin through a nasogastric tube, analyzed 1, 3, 6, 12 and 24 h after melatonin administration. Model fitting was obtained as described in Methods.

Group 1 received a single intragastric dose of 0.5 mg·kg^−1^ melatonin, and the pharmacokinetic profile was analyzed up to 24 h. As shown in Table 2, the *C*_max_ was 0.44 ± 0.06 µg·mL^−1^, and was reached in 4.30 ± 0.68 h (*T*_max_; range 2.70–6.30; Table 2 and Figure 1). Using one compartment model analysis, we found a *T*_1/2_ (elimination half-life) of 10.94 ± 1.58 h, a CL (mean apparent clearance) of 31.19 ± 5.64 L·h^−1^, and an AUC of 10.48 ± 2.09 μg·mL^−1^·h.

Group 2 and Group 3 received melatonin at the dose of 1 and 5 mg·kg^−1^, respectively. The pharmacokinetic profile was analyzed up to 24 h. These patients also received two additional doses of melatonin (i.e., 24 and 48 h after the first administration) after taking the 24 h blood sampling. As reported in Table 3, the administration of a single bolus of 1 mg·kg^−1^ melatonin resulted in a *C*_max_ of 1.03 ± 0.27 µg·mL^−1^, which was reached in 2.91 ± 1.08 h (*T*_max_). One compartment model analysis gave a *T*_1/2_ of 9.37 ± 4.25 h, an AUC of 22.26 ± 7.19 μg·mL^−1^·h and a CL of 94.93 ± 54.57 L·h^−1^.

The pharmacokinetic parameters obtained after administration of 5 mg·kg^−1^ melatonin are reported in Table 4. In this group, the *C*_max_ was 7.04 ± 1.50 µg·mL^−1^, and was reached in 4.70 ± 1.37 h (*T*_max_). The one compartment model analysis gave a *T*_1/2_ of 7.98 ± 1.45 h, a CL of 61.03 ± 12.54 L·h^−1^ and an AUC of 118.77 ± 33.02 μg·mL^−1^·h (Table 4). Figure 1 shows the plasma melatonin concentrations versus time for all groups.

**Figure 2:**
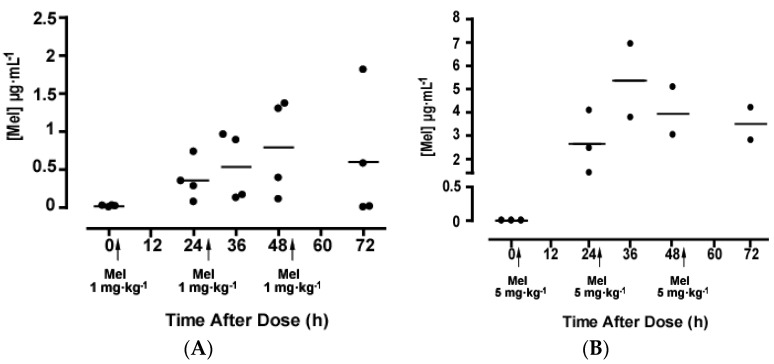
Plasma melatonin concentrations in preterm neonates after repeated melatonin administration. Melatonin was administered at a dose of 1 mg·kg^−1^ (**A**) or 5 mg·kg^−1^ (**B**), and the administration was repeated after 24 and 48 h. Plasma melatonin concentrations were measured immediately before (*T* = 0) and 24, 36, 48 and 72 h after the first administration.

As mentioned above, Group 2 and Group 3 received two additional doses of melatonin at 24 and 48 h. Blood samples were collected at 36, 48 and 72 h. Twenty-four hours after the first administration of 1 mg·kg^−1^ melatonin (Group 2), the residual concentration was 0.35 ± 0.13 µg·mL^−1^. After administration of the second dose, plasma melatonin concentration increased to 0.53 ± 0.22 µg·mL^−1^ and to 0.79 ± 0.31 µg·mL^−1^, at 12 and 24 h, respectively. The residual serum melatonin concentration measured at 24 h after the third administration (72 h after the first one) was 0.60 ± 0.42 µg·mL^−1^ (Figure 2A). 

Twenty-four hours after the first administration of 5 mg·kg^−1^ melatonin (Group 3), the residual melatonin concentration was 2.65 ± 0.77 µg·mL^−1^. Plasma concentration increased to 5.36 ± 1.58 µg·mL^−1^ at 12 h from the administration of the second dose of melatonin, and was 3.94 ± 1.03 µg·mL^−1^ after 24 h. The residual serum melatonin concentration measured at 72 h, 24 h after the third dose, was 3.51 ± 0.70 µg·mL^−1^ (Figure 2B).

## 3. Discussion

Clinical studies have proven the therapeutic benefits of melatonin in different fields of medicine, in both adults [29] and neonates [19,20,21,22,24]. In addition, melatonin appears safe, since no side effects have been reported, even with doses up to 100 mg/kg administered in 54 h [30], or when a dose of 10 mg/kg was administered once daily for 5 days [22].

Studies performed in experimental animals [8,9,10,12,13] and humans [19,22,30] indicate that the protective effect of melatonin occurs at doses ranging from 5 to 15 mg·kg^−1^. These doses elicit much higher blood melatonin concentrations and AUC values when compared with the physiological values, which show species and age differences. A dose of 10 mg·kg^−1^, for example, resulted in AUC values of 2.49 μg·mL^−1^ h in rats, 3.44 μg·mL^−1^·h in dogs, and 8.85 μg·mL^−1^·h in monkeys [26]. In addition, melatonin pharmacokinetics also show age differences, as highlighted in the recent study of Merchant et al. [28], who reported a half-life of 15.82 h after 2 h infusion of a low dose of melatonin (0.1 µg·kg^−1^·h^−1^). This half-life value is much higher compared with that found in adults (about 60 min [25]).

We report here, for the first time, the pharmacokinetic profile of oral pharmacological doses of melatonin in preterm neonates. We used three different doses of melatonin: 0.1, 0.5 and 5 mg·kg^−1^. These doses were extrapolated according to allometric evaluations [31], and on the safety demonstrated by melatonin in neonates with respiratory distress syndrome, bronchopulmonary dysplasia and sepsis [20,21,32]. The results clearly show that a single intragastric administration of these pharmacological doses to neonates born before the 37th week of gestation resulted in high peak plasma concentrations and AUC values. After administration of a single 0.5 mg·kg^−1^ intragastric bolus, blood melatonin resulted in the high nM range, and reached the μM range after administration of both 1 mg·kg^−1^ and 5 mg·kg^−1^. Although the high AUC values in preterm neonates indicate that melatonin is well absorbed after the intragastric bolus, the *C*_max_ was reached only after 4.30, 2.91, and 4.70 h (*T*_max_), for Group 1, 2 and 3, respectively. These *T*_max_ values are higher compared to adults, and do not appear to depend on the dose, differently from what is reported in adults [33]. In premature neonates, in addition, melatonin showed a prolonged elimination half-life. We found a *T*_1/2_ of 11, 9, and 7 h after the doses of 0.5, 1 or 5 mg·kg^−1^ melatonin, respectively, much higher compared to what was found after oral administration in adults [25], and in line with the results of Merchant et al. [28] after melatonin infusion in preterm infants. 

It has been previously shown that neonates born before the 31st week of gestation had low to undetectable plasma melatonin concentrations [28]. In our study, we found basal levels of melatonin in 13 out of the 15 preterm enrolled infants. For two infants, blood samples at Time point 0 (T0) were not available (see the Results section). Basal levels were in the pM range, and showed marked differences among subjects (4–506 pg·mL^−1^). The higher basal melatonin concentrations found in our study compared with the study of Merchant et al. [28] may reflect the different method used to assess melatonin (LC-MS/MS vs radioimmunoassay). With our method, indeed, we detected not only the free amount of melatonin in plasma, but also the amount bound to plasma proteins. Since it has been suggested that, at least in adults, 61–78% of melatonin can be reversibly bound to albumin [34], we can approximately estimate that the amount of free melatonin may range between 1.2–151.8 pg·mL^−1^. However, it should be considered that when compared to adults, neonates at birth have lower plasma concentrations of both albumin and α1-acid glycoprotein [35,36]. Furthermore, neonates also have higher circulating bilirubin and free fatty acids, which can displace drugs from albumin binding sites [37]. Thus, it is conceivable that the same dose of melatonin in adults and neonates may result in an increased plasma concentration of unbound melatonin in the latter.

Another interesting finding of the present study is the prolonged half-life of melatonin in premature neonates compared to adults. In adults, melatonin is mainly metabolized in the liver to 6-OH melatonin and excreted as sulfate (70%) and glucuronide (6%) conjugates [31], whereas less than 1% of circulating melatonin is excreted unchanged into urine [38]. Endogenous plasma melatonin correlates with urinary melatonin and its primary metabolite, 6-sulfatoxymelatonin, although this correlation does not seem to be absolute [39]. In the present study, we did not measure the level of excretion of melatonin and 6-sulfatoxymelatonin in the urine. This represents a shortcoming of the present study, because the most plausible explanation for the different pharmacokinetics of melatonin in adults and premature neonates may be the differences in the metabolic and elimination rates of melatonin. Indeed, it should be considered that metabolic and elimination functions are immature in neonates, and could be worse in preterm neonates. Newborns also have a limited capacity for hepatic biotransformation; in general, cytochrome P450 enzyme-mediated metabolism improves with postnatal age, and generally approaches adult levels only after the first year of life [40]. In particular, the cytochrome isoform CYP1A2, that is required for melatonin hydroxylation at the C-6 position, has been shown to have negligible activity before birth [41], and adult metabolism patterns are seen no earlier than 7–8 months of age [42]. In newborns, conjugation reactions are also inefficient, and result in a reduced ability to eliminate both exogenous and endogenous compounds [43]. In addition to these physiological features, the pharmacological doses used in our study may largely exceed the metabolic capacity of the developing liver, allowing the reabsorption of the lipophilic unmodified melatonin in the kidney. All these factors, along with the observation that nephrogenesis in preterm neonates is incomplete and could compromise glomerular and tubular function [44], could explain the pharmacokinetic parameters observed in this study.

## 4. Materials and Methods

### 4.1. Study Population

The study was designed and conducted in accordance with the Declaration of Helsinki, and was approved by the Ethics Committee of the University Hospital of Messina (approval number E41/13). Prior to the study, written informed consent was obtained from the parents. Fifteen premature newborns admitted to the Neonatal Intensive Care Unit of the University Hospital of Messina, Italy, were enrolled in the study within 24–48 h after birth. Newborns were fed with formula milk when mother’s milk was not available, starting 12 h after birth. Melatonin was administrated 1 h after feeding. Inclusion and exclusion criteria of the enrolled newborns were as follows:

(a) inclusion criteria: gestational age <37 weeks; normal liver function test (i.e., serum bilirubin, alkaline phosphatase, serum glutamic-oxaloacetic transaminase, serum glutamic pyruvic transaminase, etc.), normal kidney function test (i.e., serum creatinin levels, blood urea nitrogen); presence of an indwelling vascular catheter;
(b) exclusion criteria: obvious congenital malformation; oliguria (<1 mL·kg^−1^·h^−1^) during preceding eight hours; documented infection. Demographic and clinical data are reported in Table 1.

### 4.2. Dose and Medication

Using a computer-generated randomization schedule, newborns were randomly assigned to three different groups. Group 1 received a single intragastric bolus of 0.5 mg·kg^−1^ melatonin, whereas Group 2 and Group 3 received 3 intragastric boluses of 1 or 5 mg·kg^−1^ melatonin, respectively, at 24-h intervals.

Melatonin (Melamil^®^ 5 mg·mL^−1^; Milte Italia SpA, Milan, Italy) was prepared by the hospital pharmacist according to the randomization assignment and administered by a nurse through a nasogastric tube. After administration, the tube was flushed with 0.5 mL of sterile water to ensure the full delivery of melatonin.

To determine the pharmacokinetic parameters, blood samples (0.5 mL) were collected through an indwelling arterial catheter immediately before (time 0) and 1, 3, 6, 12, 24, 36, 48 and 72 h after melatonin administration. Samples were collected in plastic tubes without anticoagulant agents. The serum was immediately separated by centrifugation, and stored at −20 °C until assayed. Research staff remained unaware of groups’ assignment until the completion of data analysis.

### 4.3. Melatonin Concentration Assay

Plasma melatonin concentration was measured according to the method of Wang et al. [45]. Briefly, stock solutions of melatonin (1.00 mg·mL^−1^; 99.9% Sigma Aldrich SRL, Milano, Italy) and *N*-acetyltryptamine (1.00 mg·mL^−1^; Sigma Aldrich SRL, Milano, Italy) were used as internal standards. These solutions were prepared in acetonitrile (Sigma Aldrich SRL, Milano, Italy) and stored at −20 °C for a maximum of 2 weeks. On the day of analysis, a dilution of the internal standard stock solutions was prepared in water (Millipore Direct-Q5; Merek S.p.a., Vimodrone, Italy) to give a working concentration of about 20 ng·mL^−1^. Working standard solutions of melatonin were prepared by serial dilutions of the stock solution.

Calibration, quality control (QC), or unknown samples were added to heat-treated glass tubes in a final volume of 550 μL. After the addition of 50 μL *N*-acetyltryptamine working solution and 50 μL ammonium hydroxide (10% in water) (Fluka, Milano, Italy), samples were briefly shaken. Two mL dichloromethane (Fluka, Milano, Italy) was added, and samples vortexed at 600 rpm for 5 min. Tubes were then centrifuged at 4 °C and 3000× *g* for 10 min (Eppendorf Centrifuge 5810R, Hamburg, Germany), the lower organic phase transferred to another heat-treated glass tube and evaporated to dryness under nitrogen stream at room temperature. The residue was reconstituted in 100 μL water by vortex mixing at 600 rpm for 2 min. After centrifugation at 3000× *g* for 5 min, the supernatant was transferred into heat-treated glass autosampler vial inserts.

High-performance liquid chromatography (HPLC) and mass spectrometry (MS/MS) analyses were performed on an Agilent Technologies 1200 series system and an ABSciex API 4000 triple-quadrupole mass spectrometer, respectively.

The HPLC was equipped with a G1322A degasser (Agilent Technologies International Sarl, Morges, Switzerland), a G1312B SL binary pump (Agilent Technologies International Sarl, Morges, Switzerland), and a G1316B SL thermostatted column compartment (Agilent Technologies International sarl, Morges, CH, Switzerland) equipped with an Agilent ZORBAX Eclipse XDB C18 column (4.6 mm i.d. × 150 mm, 5 μm) (Agilent Technologies International Sarl, Morges, Switzerland) and a Phenomenex Security-Guard C18 guard column (4 mm × 3.0 mm i.d.) (Phenomenex Inc., Torrance, CA, USA). The injection volume was 10 μL. Before injecting, the injector needle was washed with 100 μL of both methanol–water (20:80) and methanol–water (80:20). The washing procedure was repeated five times after injection. Samples were gradient-eluted at 0.5 mL·min^−1^ using 2 mM ammonium formate/0.1% formic acid (Fluka, Milano, Italy) in water and acetonitrile. The total run time was 11 min.

The mass spectrometer was operated with an Agilent G1948B ionization source (Applied Biosystem, Concord, ON, Canada) in positive ESI mode. The ABSciex Analyst software (Applied Biosystem, Concord, ON, Canada) for equipment control, data acquisition, and analysis was used. The MS/MS parameters for melatonin and *N*-acetyltryptamine were manually optimized by separately infusing standard solutions. Accordingly, the instrument was operated with the capillary voltage at +4.0 kV, and nozzle voltage at +500 V. Nitrogen was used as a nebulizer gas at 20 psi, as carrier gas at 11 L·min^−1^ at 350 °C, and as sheath gas at 7 L·min^−1^ at 250 °C. The multiple reaction monitoring (MRM) was employed for data acquisition. The optimized MRM fragmentation transitions were: (i) melatonin, *m*/*z* 233.1 → *m*/*z* 174 with a fragmentor voltage of 110 V and collision energy (CE) of 9 V; (ii) *N*-acetyltryptamine, *m*/*z* 203.1 → *m*/*z* 144 with a fragmentor voltage of 80 V and CE of 9 V.

### 4.4. Pharmacokinetic Analysis

The pharmacokinetic parameters of melatonin were individually estimated. We used one compartment model for extravascular input, with no lag time, and first order elimination, as described by the following equation (Concentration vs. time):

(1) $$C\left(T\right)=A\left(e^{-K_{\mathrm{el}}T}-e^{-K_{a}T}\right)$$

where *A* is a constant factor, *K*_el_ is the elimination constant, *K*_a_ is the absorption constant. Model fitting was obtained using the minimization approach, according to the Gauss–Newton method, and PKSolver software [46].

In addition, a software developed in the Microsoft Excel environment was used to estimate the following pharmacokinetic parameters:

(i) elimination half-life (*T*_1/2_)
  (2) $$T_{\frac{1}{2}}=\frac{\ln2}{K_{\mathrm{el}}}$$
(ii) time of peak concentration (*T*_max_)
  (3) $$T_{\max}=\frac{\frac{\ln\;K_{a}\;}{K_{\mathrm{el}}}}{K_{a}-K_{\mathrm{el}}}$$
(iii) maximal concentration (*C*_max_)
  (4) $$C_{\max}=A\left(e^{-K_{\mathrm{el}}T_{\max\;}}-e^{-K_{a}T_{\max\;}}\right)$$
(iv) area under the curve (*AUC*)
  (5) $$AUC=\int_{0}^{\infty }Cdt$$
(iv) area under the first moment curve (*AUMC*)
  (6) $$AUMC=\int_{0}^{\infty }tCdt$$
(vi) mean residence time (*MRT*)
  (7) $$MRT=\frac{AUMC}{AUC}$$

Outliers has been treated according to the Dixon test [47].

## 5. Conclusions

In conclusion, this study provides new fundamental data on the pharmacokinetic profile of oral pharmacological doses of melatonin in preterm infants. Results point out the possibility to obtain and maintain neuroprotective concentrations of melatonin in blood using a single oral administration of the indoleamine repeated every 12/24 h. Because melatonin is proposed as adjunctive therapy to hypothermia for perinatal ischemic and inflammatory brain injuries [48], data from this study can be helpful to prescribe proper dosage and frequency of administration for this particular population.

## Figures and Tables

**Table 1:** Demographic and clinical data.

Demographic and Clinical Data	Group-1		Group-2		Group-3	
	Mean	Range	Mean	Range	Mean	Range
Age (weeks)	31	26–33	33	27–36	30	28–33
Sex (M/F)	3:3		4:0		1:4	
Birth Weight (g)	1461	780–2150	2185	1050–3200	1803	1100–2830
Apgar 1’	7	4–8	7	5–8	8	8–9
Apgar 5’	8	7–9	9	8–9	9	8–9
MV ^1^ (h)	14	0–98	18	0–120	0	0

^1^ MV, mechanical ventilation.

**Table 2:** Pharmacokinetic parameter estimates of melatonin in Group-1.

Patient	A	B	C *	D	E	F	Mean ± SE
*K*_el_	0.06	0.07	0.05	0.11	0.04	0.05	0.07 ± 0.01
*K*_a_	0.07	0.36	0.28	0.55	0.38	1.20	0.51 ± 0.19
*T*_1/2_ (h)	10.51	9.44	12.35	6.20	15.51	13.03	10.94 ± 1.58
AUC (μg·mL^−1^·h)	7.74	15.27	0.307	4.12	14.54	10.75	10.48 ± 2.09
AUMC (μg·mL^−1^·h)	203.03	367.01	8.90	67.19	460.67	251.90	269.96 ± 31.19
*T*_max_ (h)	3.40	5.50	7.2	3.60	6.30	2.70	4.30 ± 0.68
*C*_max_ (µg·mL^−1^)	0.32	0.58	0.008	0.30	0.40	0.59	0.44 ± 0.06
MRT (h)	26.20	24.03	28.97	16.29	31.66	23.41	24.32 ± 2.48

*K*_el_, elimination constant; *K*_a_, adsorption constant; *T*_1/2_, elimination half-time; AUC, Area Under concentration–time Curve; AUMC, Area Under the first Moment Curve, *T*_max_, time to reach maximal serum concentration; *C*_max_, maximal serum concentration; MRT, Mean Residence Time; * Mean and standard error were calculated excluding patient C according to the Dixon test (*p* < 0.01).

**Table 3:** Pharmacokinetic parameter estimates of melatonin in Group-2.

Patient	G	H	I	L	Mean ± SE
*K*_el_	0.03	0.16	0.36	0.06	0.15 ± 0.07
*K*_a_	2.08	0.84	1.83	2.75	1.88 ± 0.40
*T*_1/2_ (h)	20.81	4.07	1.88	10.73	9.37 ± 4.25
AUC (μg·mL^−1^·h)	37.58	28.54	3.95	18.96	22.26 ± 7.19
AUMC (μg·mL^−1^·h)	1386.85	348.18	31.42	344.95	527.85 ± 295.81
*T*_max_ (h)	2.25	6.10	1.88	1.40	2.91 ± 1.08
*C*_max_ (µg·mL^−1^)	0.88	1.78	0.50	0.97	1.03 ± 0.27
MRT (h)	36.89	12.19	7.94	18.18	18.80 ± 6.39

*K*_el_, elimination constant; *K*_a_, adsorption constant; *T*_1/2_, elimination half-time; AUC, Area Under concentration–time Curve; AUMC, Area Under the first Moment Curve, *T*_max_, time to reach maximal serum concentration; *C*_max_, maximal serum concentration; MRT, Mean Residence Time.

**Table 4:** Pharmacokinetic parameter estimates of melatonin in Group-3.

Patient	M	N	O	P	Q	Mean ± SE
*K*_el_	0.13	0.12	0.06	0.11	0.06	0.09 ± 0.01
*K*_a_	0.67	0.61	2.83	0.11	0.14	0.87 ± 0.50
*T*_1/2_ (h)	5.19	5.66	11.45	6.03	11.58	7.98 ± 1.45
AUC (μg·mL^−1^·h)	53.87	89.21	245.24	94.66	110.59	118.77 ± 33.02
AUMC (μg·mL^−1^·h)	787.32	1411.78	4300.18	1100.50	2063.28	1932.61 ± 628.40
*T*_max_ (h)	3.01	3.30	1.40	8.70	7.10	4.70 ± 1.37
*C*_max_ (µg·mL^−1^)	3.54	5.20	12.25	8.21	6.02	7.04 ± 1.50
MRT (h)	14,61	15.82	17.53	11.62	18.65	15.64 ± 1.22

*K*_el_, elimination constant; *K*_a_, adsorption constant; *T*_1/2_, elimination half-time; AUC, Area Under concentration–time Curve; AUMC, Area Under the first Moment Curve, *T*_max_, time to reach maximal serum concentration; *C*_max_, maximal serum concentration; MRT, Mean Residence Time.
